# An autism-associated mutation in Ca_V_1.3 channels has opposing effects on voltage- and Ca^2+^-dependent regulation

**DOI:** 10.1038/srep27235

**Published:** 2016-06-03

**Authors:** Worawan B. Limpitikul, Ivy E. Dick, Manu Ben-Johny, David T. Yue

**Affiliations:** 1 Calcium Signals Laboratory, Departments of Biomedical Engineering and Neuroscience, The Johns Hopkins University School of Medicine, Ross Building, Room 713,720 Rutland Avenue, Baltimore, MD 21205, USA.

## Abstract

Ca_V_1.3 channels are a major class of L-type Ca^2+^ channels which contribute to the rhythmicity of the heart and brain. In the brain, these channels are vital for excitation-transcription coupling, synaptic plasticity, and neuronal firing. Moreover, disruption of Ca_V_1.3 function has been associated with several neurological disorders. Here, we focus on the *de novo* missense mutation A760G which has been linked to autism spectrum disorder (ASD). To explore the role of this mutation in ASD pathogenesis, we examined the effects of A760G on Ca_V_1.3 channel gating and regulation. Introduction of the mutation severely diminished the Ca^2+^-dependent inactivation (CDI) of Ca_V_1.3 channels, an important feedback system required for Ca^2+^ homeostasis. This reduction in CDI was observed in two major channel splice variants, though to different extents. Using an allosteric model of channel gating, we found that the underlying mechanism of CDI reduction is likely due to enhanced channel opening within the Ca^2+^-inactivated mode. Remarkably, the A760G mutation also caused an opposite increase in voltage-dependent inactivation (VDI), resulting in a multifaceted mechanism underlying ASD. When combined, these regulatory deficits appear to increase the intracellular Ca^2+^ concentration, thus potentially disrupting neuronal development and synapse formation, ultimately leading to ASD.

L-type voltage-gated Ca^2+^ channels are critical conduits for Ca^2+^ entry into many excitable cells. The Ca_V_1.3 channel represents a distinctive subtype of these channels, important in neurological^1^,^2^,^3^,^4^, cardiac^3^,^4^,^5^, and endocrine^4^,^6^,^7^ function. The biophysical properties of these channels are thus precisely tuned to this function, as they are activated at relatively hyperpolarized potentials compared to other L-type voltage-gated Ca^2+^ channels^3^,^8^,^9^,^10^,^11^,^12^ and undergo distinct forms of negative feedback regulation^3^,^13^,^14^.

Ca_V_1.3 channels employ two major forms of feedback regulation, voltage-dependent inactivation (VDI) and Ca^2+^-dependent inactivation (CDI)^14^. These two regulatory processes are controlled within each cell type, utilizing splice variation^3^,^15^,^16^,^17^, RNA editing^18^,^19^, and auxiliary subunit pairing^20^,^21^ to tune the inactivation properties of the channel to specific cellular functions. In particular, both splice variation and RNA editing are able to modulate both CDI^3^,^10^,^17^,^18^,^19^,^22^,^23^,^24^ and channel open probability^15^ by tailoring the components contained within the channel carboxy tail. In addition, channel beta subunits are known to both traffic channels to the membrane^25^,^26^ and alter their voltage inactivation properties^21^,^26^,^27^,^28^.

The precise control of these regulatory processes are a vital component of normal physiology and disruption of this regulation has been linked to multiple human disorders including autism^3^,^29^,^30^,^31^, auditory deficits^32^,^33^, and hyperaldosteronism^34^,^35^. In mice, knockout of Ca_V_1.3 results in profound deafness and severe bradycardia^33^,^36^, while in humans a similar phenotype is observed in patients harboring a 3-base pair insertion in exon 8b^32^. This insertion abolishes channel conduction, resulting in sinoatrial node dysfunction and deafness (SANDD) syndrome, a phenotype similar to that described in Ca_V_1.3-knockout mice. Moreover, multiple gain-of-function mutations have been linked to patients with hyperaldosteronism^34^,^35^. Finally, two gain-of-function mutations in Ca_V_1.3 (G407R and A749G) have been linked to autism spectrum disorders (ASD)^30^,^31^,^37^. Prior studies of these two mutations demonstrated alterations in channel gating including a hyperpolarizing shift in channel activation and inactivation curves^31^, but the differential effects on CDI versus VDI have yet to be determined. Discerning these precise effects may be highly relevant to understanding the mechanism of pathogenesis, as disruption of each of these components in the related Ca_V_1.2 L-type channel has been shown to underlie Timothy syndrome (a severe multisystem disorder including autism and cardiac deficits)^38^,^39^,^40^, as well as long-QT syndrome associated with mutations in calmodulin^41^. It is interesting to note that, unlike the Ca_V_1.2 channelopathies, Ca_V_1.3 mutations have often been associated with single-system phenotypes^30^,^37^, despite the multi-system distribution of Ca_V_1.3 channels. This isolation of symptoms is curious and requires further mechanistic investigation.

Here, we examine the underlying channel regulatory deficits of the autism-associated A760G mutation in rat Ca_V_1.3 (equivalent to the A749G^31^ or A769G^30^ mutation in the human, depending on the channel backbone), focusing on the specific biophysical alterations produced by the mutation. We find that the mutation causes a significant reduction of CDI and a delay in channel deactivation in two major channel splice variants. In addition, we utilize an allosteric model of channel gating to gain insight into the underlying mechanism of this CDI deficit. Further examination of the biophysical defects of this mutation also revealed a beta subunit-dependent increase in VDI, an effect which would oppose the Ca^2+^ overload due to the decrease in CDI and a delay in channel deactivation. Thus the severe effects of this ‘gain-of-function’ mutation could be mitigated by a ‘loss-of-function’ effect on VDI.

## Results

### A760G significantly decreases CDI and alters Ca_V_1.3 channel gating

**Figure 1:**
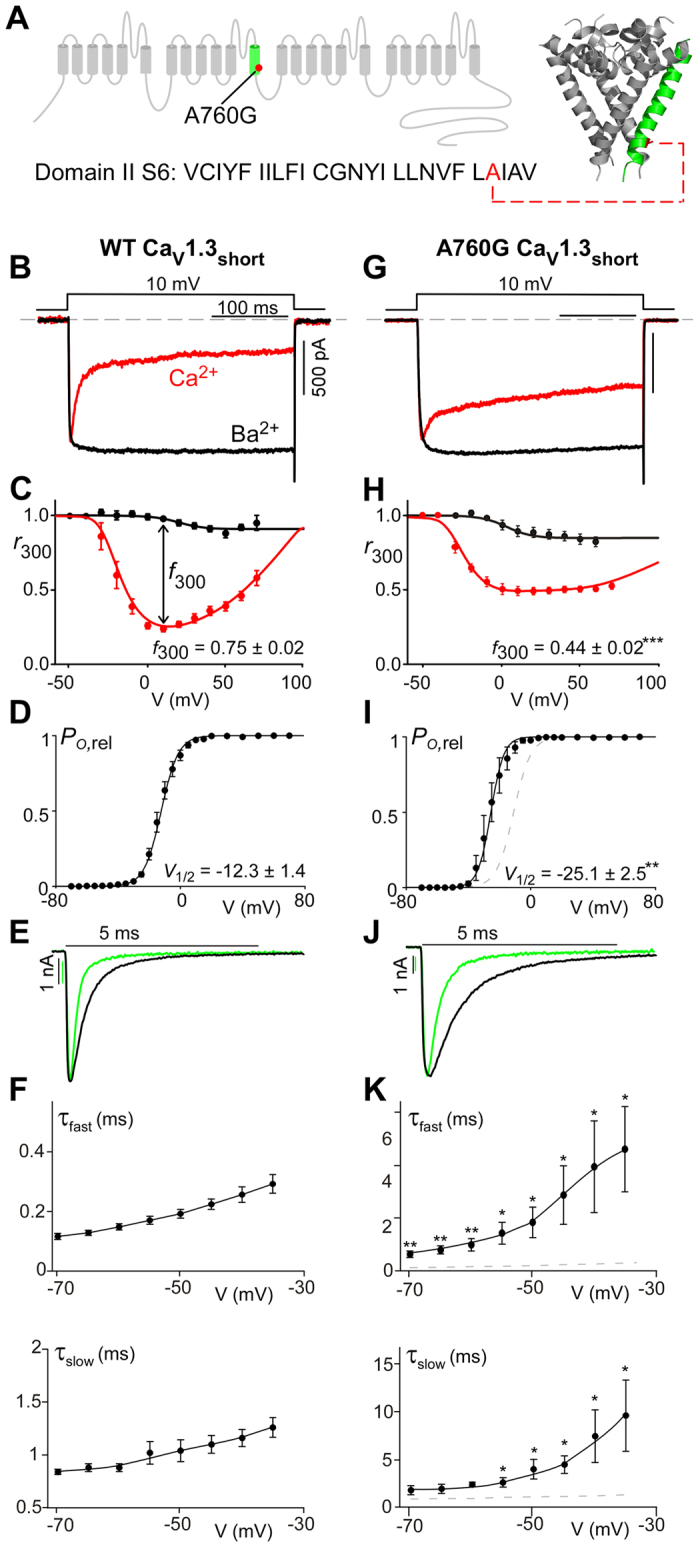
A760G decreases CDI and promotes channel activation. (**A**) Cartoon representing Ca_v_1.3 and the location of the autism-associated mutation A760G. Left, the A760G missense mutation resides in the S6 helix of domain II. Right, structural homology model of Ca_v_1.3 S5/S6 segments, based on Na_V_Ab with the A760G highlighted in red. (**B**) Exemplar Ca^2+^ (red) and Ba^2+^ (black) current traces through WT Ca_V_1.3_short_ evoked by a 10-mV depolarizing step. Currents are normalized for comparison. Scale bar corresponds to the Ca^2+^ trace. (**C**) Population data of fraction of current remaining after 300 ms (*r*_300_) for Ca^2+^ (red) and Ba^2+^ (black). *f*_300_ determined at 10 mV (n = 12). Data are plotted as mean ± SEM here and throughout. (**D**) The activation curve for WT Ca_V_1.3_short_ obtained via a tail activation protocol with Ba^2+^ as the charge carrier (n = 6). (**E**) Exemplar Ba^2+^ tail currents obtained from a transition from 80 mV to −40 mV (black) and −60 mV (green). Traces are normalized to one another. Scale bars correspond to the traces of the same color. (**F**) Population data of the fast (top) and slow (bottom) deactivation time constants (τ) plotted as a function of voltage (n = 5). (**G**) Exemplar Ca^2+^ (red) and Ba^2+^ (black) current traces through A760G Ca_V_1.3_short_ evoked by a 10-mV depolarizing step. Compared to that of WT (**B**), Ca^2+^ current through A760G channels display significantly less CDI. (**H**) Population data of *r*_300_ for Ca^2+^ (red) and Ba^2+^ (black) currents through A760G channels. CDI is significantly smaller (***p < 0.001, n = 6) than that of the WT channels. (**I**) The activation curve of A760G Ca_V_1.3_short_ channels (black) shows a 13-mV hyperpolarizing shift (**p < 0.01, n = 4) compared to WT (reproduced in gray). (**J**) Exemplar Ba^2+^ tail currents for the A760G channel, obtained from a transition from 80 mV to −40 mV (black) and −60 mV (green). (**K**) Population data of the fast (top) and slow (bottom) deactivation time constants (τ) plotted as a function of voltage for the A760G channel. Deactivation is significantly slowed as compared to WT reproduced as the gray dashed line (*p < 0.05, **p < 0.01, n = 4).

Voltage-gated Ca^2+^ channel α1-subunits are composed of four domains, each containing six transmembrane α -helices (Fig. 1A). The four S6 helices line the channel pore through which Ca^2+^ enters the cell. The intracellular portion of these S6 helices form the activation gate of the channel, and mutations within this region are known to alter channel activation^31^,^42^,^43^,^44^,^45^,^46^. Moreover, the S6 helices are known to contribute to VDI and CDI in many Ca_V_ channels, including Ca_V_1.3^15^,^40^,^42^,^43^,^44^,^45^,^46^,^47^,^48^,^49^,^50^. Thus, the effect of the A760G mutation on channel activation and inactivation^31^ may be explained by its location in the S6 helix of domain II (IIS6).

In order to study the effects of the A760G mutation on Ca^2+^ regulation of channels, we undertook whole-cell voltage clamp recordings of Ca_V_1.3 channels heterologously expressed in HEK293 cells. To minimize the possible confounding effects on CDI measurements, the channels were co-expressed with the β_2a_ subunit which is known to minimize VDI^21^,^26^,^28^,^51^,^52^. In addition, an internal solution containing 10 mM BAPTA was utilized to restrict Ca^2+^ elevation to only the nanodomain of the channel, thus diminishing cell-to-cell variability^53^,^54^. Figure 1B shows exemplar current traces for wild-type (WT) Ca_V_1.3. The sharp decay of Ca^2+^ current (red) evoked by a 10-mV depolarizing step depicts CDI, while the Ba^2+^ current trace (black) exhibits only VDI, which is mostly absent here due to the choice of β subunit. This robust CDI can be observed in the population data, where the fraction of current remaining after 300-ms depolarization (*r*_300_) is displayed as a function of voltage (Fig. 1C), and the U-shaped dependence on voltage recapitulates a classic hallmark of CDI^55^,^56^. Here, the difference between the *r*_300_ values for Ca^2+^ and Ba^2+^, normalized by the Ba^2+^ *r*_300_, gauges the magnitude of CDI (*f*_300_). However, when the A760G mutation is introduced into these channels, a drastic reduction in the speed and magnitude of CDI is observed (Fig. 1G, Supplementary Figure 1). This effect is further evident in the population data across multiple voltages (Fig. 1H) illustrating a clear CDI deficit due to the autism-associated A760G mutation.

Beyond CDI, S6 mutations are likely to affect channel activation^31^,^42^,^43^,^44^,^45^,^46^. We therefore probed the effect of the A760G on the voltage dependence of Ca_V_1.3 channel activation. Here, we measured the relative open probability (*P*_o,rel_) of the channels across voltages via a tail activation protocol optimized for Ca_V_1.3 channels^54^ (Fig. 1D). Introduction of the A760G mutation produced a significant hyperpolarizing shift (13 mV; p < 0.01) in channel activation (Fig. 1I), consistent with previous studies of this channel^31^. Moreover, analysis of the deactivation kinetics during the tail activation protocol revealed a marked effect of A760G on channel deactivation. Evaluation of the deactivation kinetics during a transitions from 80 mV to multiple voltages near the foot of the activation curve provided an estimation of the kinetics of channels transitioning from fully open to closed (Fig. 1E,F). A double exponential function was used to quantify a fast and slow component of channel deactivation (τ_fast_, τ_slow_ respectively). The A760G mutation significantly increased both time constants across voltages, indicating a considerable slowing of channel closing (Fig. 1J,K).

**Figure 2:**
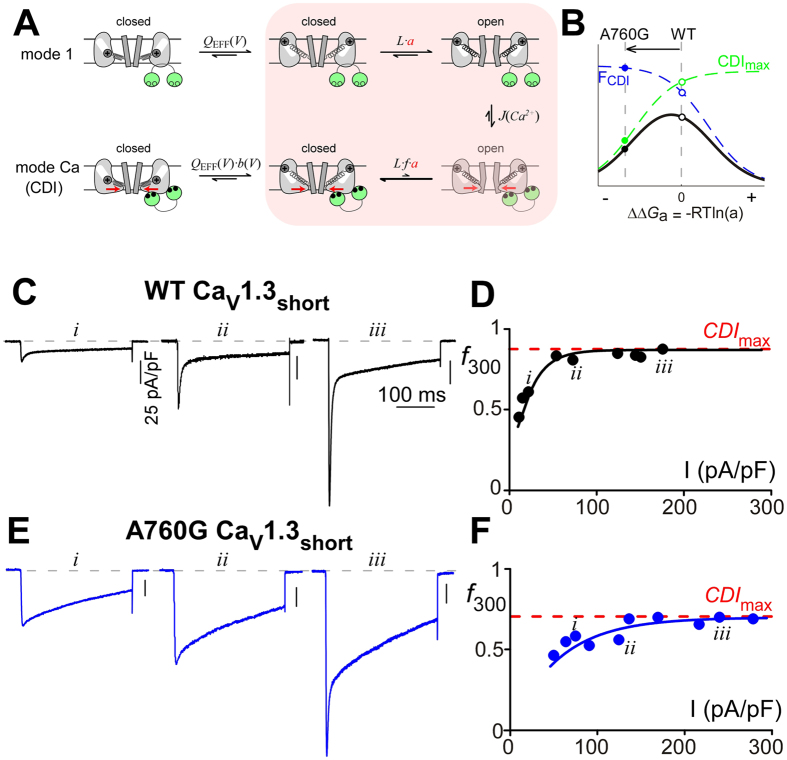
An allosteric mechanism underlying the CDI reduction. (**A**) Diagram representing an allosteric model of CDI. Channels transition from mode 1 with high *P*_O_, to mode Ca^2+^ with lower *P*_O,_ in response to Ca^2+^ entry. Equilibrium constants *Q*_*EFF*_ (concerted movement of S1–S4 segments), *L* (S6 movement), and *a* (mutation effect) govern transitions between open and closed channel configurations^14^,^38^, while the effective equilibrium constant *J*(Ca^2+^) governs entry into mode Ca^2+^. The parameter *f* (0 < *f* < 1) scales the *P*_O_ in mode Ca^2+^ resulting in CDI. State transitions expected to be effected by the A760G are shaded pink. (**B**) Total CDI (black), the product of F_CDI_ (blue) and *CDI*_max_ (green), is plotted as a function of ∆∆G_a_ for the model shown in panel A. The A760G mutation left-shifts voltage activation (∆∆G_a _< 0, *a* > 1), predicting a decrease in CDI due to a decrease in *CDI*_max_. (**C**) Exemplar Ca^2+^ current traces through WT Ca_V_1.3_short_ channels. Larger current amplitudes (*ii, iii*) allow a greater influx of Ca^2+^, enhancing entry into mode Ca and thus increasing CDI as compared to diminutive Ca^2+^ currents (*i*). (**D**) *f*_300_ values for individual cells expressing WT Ca_V_1.3_short_ are plotted as a function of current density. The curve saturates at *CDI*_max_ ~0.9 (red dashed line). Traces in C correspond to *i*–*iii*. (**E**) Exemplar Ca^2+^ current traces through A760G Ca_V_1.3_short_ channels. Similar to that of WT (**C**), larger current amplitude increases *f*_300_ values. (**F**) Population data representing CDI of A760G Ca_V_1.3_short_ channels. *f*_300_ values saturate at *CDI*_max_ ~0.7 (red dashed line), significantly lower than WT.

L-type channel S6 mutations have previously been shown to affect CDI via modulation of modal channel gating^14^,^38^,^57^. We therefore consider the underlying mechanism linking channel activation and CDI. An allosteric model of channel gating (Fig. 2A) is known to describe CDI of Ca_V_1.3 channels well^14^. Within this model, channels initially open within the mode 1 regime, which is characterized by a relatively large open probability (*P*_O/mode1_). Upon channel opening, Ca^2+^ influx drives channels into the mode Ca regime where channels maintain the ability to open, but with a significantly reduced *P*_O_ (*P*_O/modeCa_). It is this reduction in *P*_O_ that results in the CDI seen in whole-cell currents, such that


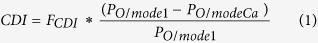


where *F*_CDI_ is the fraction of channels within mode Ca. Therefore at a saturating level of Ca^2+^, *F*_CDI_ will approach unity as virtually all channels will reside within mode Ca. Under this condition, a maximal level of CDI (*CDI*_max_) is achieved:


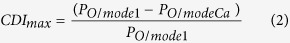


We now consider the effects of the S6 mutation A760G within our model. As we and others^31^ have shown that this mutation causes a hyperpolarizing shift in channel activation, the variable *a* was introduced to account for the altered free energy (∆∆G_a_) required to open the mutant channels^14^. For a hyperpolarizing mutation such as A760G, ∆∆G_a_ will be negative, indicating decreased energy required to open the mutant channel. This decrease in free energy will result in increased channel opening in both mode 1 and mode Ca, thus decreasing *CDI*_max_ (Equation 2, Fig. 2B, green). This reduction of *CDI*_max_ could account for the overall decrease in CDI (Fig. 2B, black) observed in whole-cell experiments (Fig. 1), despite the increased *F*_CDI_ (Fig. 2B, blue) due to increased Ca^2+^ influx.

To test the hypothesis that the CDI deficit due to A760G is primarily a result of a decrease in *CDI*_max_, we undertook whole-cell patch clamp recordings in which conditions enabled a sustained saturating level of Ca^2+^ at the mouth of the channel. By significantly reducing the intracellular Ca^2+^ buffer (0.5 mM EGTA), the accumulation of Ca^2+^ within the cell should overpower the nanodomain Ca^2+^ signal, thus raising Ca^2+^ to saturating levels as whole cell current increases^58^ and providing an estimation of *CDI*_max_^54^. For WT Ca_V_1.3 channels, exemplar Ca^2+^ traces illustrate a saturating amount of CDI as a function of current density (Fig. 2C,D). The lack of additional CDI accumulation beyond a current density of 50 pA/pF indicates that we reached *CDI*_max_ at a value of ~0.9 for WT channels (Fig. 2D, red dashed line). A760G channels, however, demonstrate a significant reduction in *CDI*_max_ to ~0.7 (Fig. 2E,F), which can be observed at multiple test potentials (Supplementary Figure 2), confirming the underlying mechanism of CDI loss (Fig. 2B).

### The A760G mutation differentially affects Ca_V_1.3 splice variants

Ca_V_ channels are a critical conduit for Ca^2+^ entry into multiple cell types and must therefore be precisely tuned for specific cellular functions. Nature employs multiple mechanisms with which to accomplish such fine tuning, including modulation of channel splice patterns^3^,^17^,^22^,^23^,^59^. An example of such splicing in Ca_V_1.3 channels results from inclusion of exon 42 or 42a, yielding a channel with a long versus short C-terminus^59^. The long channel variant activates at a somewhat more positive potential^3^,^24^, has a lower open probability^15^, and exhibits dramatically reduced CDI as compared to the short splice variant^3^,^16^. Due to these distinct properties, we examined the effect of the A760G mutation in both relevant splice variants. Having already demonstrated a significant reduction in CDI within the short channel variant, the isoform with more robust CDI^3^,^59^ (Fig. 1), we next undertook a similar approach within the long Ca_V_1.3 splice variant (Ca_V_1.3_long_).

**Figure 3:**
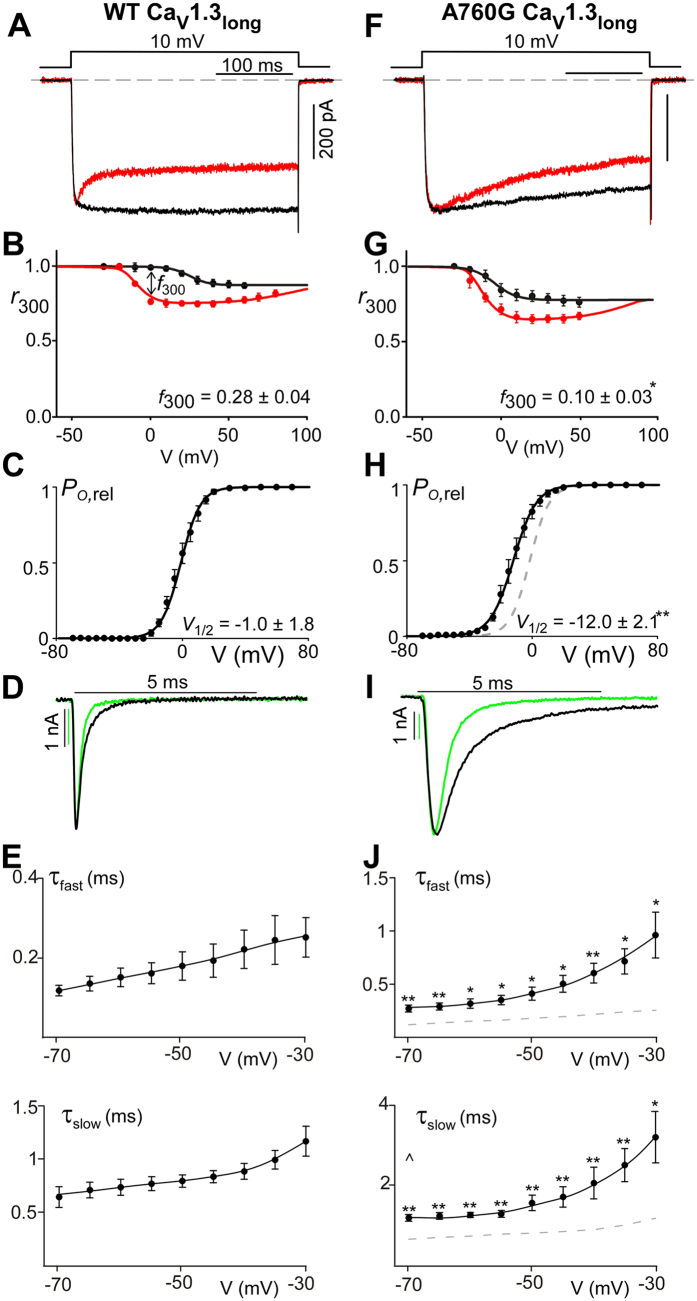
CDI reduction due to A760G mutation within the Ca_V_1.3 long variant. (**A**) Exemplar Ca^2+^ (red) and Ba^2+^ (black) current traces through the alternate splice variant Ca_V_1.3_long_ illustrating decreased CDI as compared to the short splice variant (Fig. 1B). (**B**) Despite the reduction in magnitude, significant CDI is demonstrated by the difference in *r*_300_ for Ca^2+^ (red) and Ba^2+^ (black), plotted across multiple voltages. (*f*_300_ = 0.28 ± 0.04, n = 3). (**C**) Voltage activation curve for Ba^2+^ current through WT Ca_V_1.3_long_ channels (V_1/2_ = −1.0 ± 1.8 mV, n = 5). (**D**) Exemplar Ba^2+^ tail currents obtained from a transition from 80 mV to −40 mV (black) and −60 mV (green). Traces are normalized to one another such that the scale bars correspond to the traces of the same color. (**E**) Population data of the fast (top) and slow (bottom) deactivation time constants (τ) plotted as a function of voltage. Error bars indicate ± SEM, n = 5. (**F**) Exemplar current traces through A760G Ca_V_1.3_long_ channels depicting diminished CDI as compared to WT channels. (**G**) Population data for *r*_300_ (red = Ca^2+^, black = Ba^2+^) plotted across multiple voltages (*f*_300_ = 0.10 ± 0.03; n = 4, *p < 0.05). (**H**) The activation curve of A760G Ca_V_1.3_long_ channels (black, V_1/2_ = −12.0 ± 2.1 mV, n = 4) shows an 11-mV hyperpolarizing shift (**p < 0.01) compared to WT (reproduced in gray for reference). (**I**) Exemplar Ba^2+^ tail currents for the A760G Ca_V_1.3_long_ channel, obtained from a transition from 80 mV to −40 mV (black) and −60 mV (green). Traces are normalized to one another such that the scale bars correspond to the traces of the same color. (**J**) Population data of the fast (top) and slow (bottom) deactivation time constants (τ) plotted as a function of voltage for the A760G Ca_V_1.3_long_ channel. Deactivation is significantly slowed as compared to WT reproduced as the gray dashed line (*p < 0.05, **p < 0.01). Error bars indicate ± SEM, n = 4.

Examination of CDI within the WT Ca_V_1.3_long_ channel under high buffering conditions (10 mM BAPTA) demonstrated significantly smaller, yet appreciable CDI (Fig. 3A,B), as compared to the short channel variant (Fig. 1B,C). Introduction of the A760G mutation blunted this CDI (Fig. 3F,G), though to a lesser extent as compared to the short variant. We next examined the activation of WT and A760G Ca_V_1.3_long_ channels. The resulting *P*_o,rel_ versus voltage relationships (Fig. 3C,H) demonstrated an 11 mV hyperpolarizing shift due to the introduction of A760G (p < 0.01), similar to that observed in the short variant (Fig. 1D,I). Likewise, analysis of the deactivation kinetics also revealed a significant slowing of channel closing across multiple voltages (Fig. 3D,E,I,J). Thus, the biophysical deficits produced by the A760G mutation are qualitatively similar within each relevant channel backbone, although the magnitude of the CDI effect is somewhat decreased in the long splice variant.

### Opposing VDI changes may mitigate the detrimental effects of A760G

**Figure 4:**
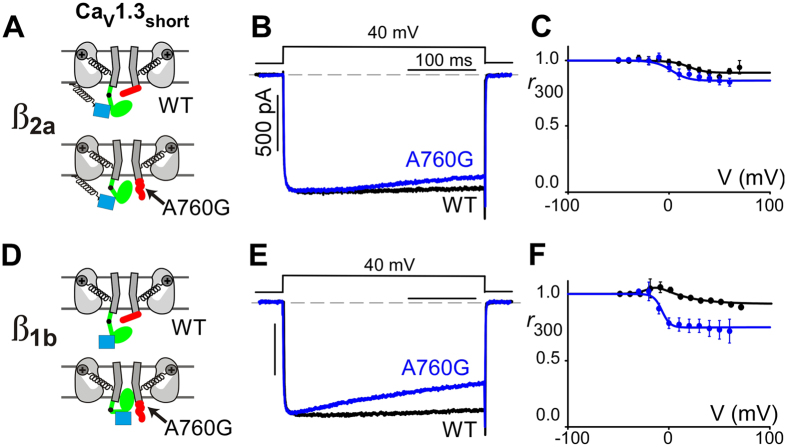
A760G increases VDI. (**A**) Cartoon depicting a β_2a_ subunit (blue) interacting with the VDI hinged lid (green) of a Ca_V_1.3_short_ channel. With palmitoylation, the β_2a_ subunit is anchored to the plasma membrane (black coil) restricting the movement of the channel hinged lid. Ca_V_1.3_short_ channels also possess a ‘VDI shield’ (red). The A760G mutation disrupts this shield (bottom). (**B**) Exemplar Ba^2+^ current through WT (black) and A760G (blue) Ca_V_1.3_short_ channels shows re-emergence of VDI even in the presence of β_2a_. (**C**) Population data displaying Ba^2+^ *r*_300_ values as a function of voltage for WT (black) and A760G (blue). At 10 mV, WT: *r*_300_ = 0.98 ± 0.01; n = 10; A760G: *r*_300_ = 0.89 ± 0.03, n = 6. VDI is significantly increased in A760G channels (p < 0.01). (**D**) Cartoon depicting a β_1b_ subunit (blue) interacting with the hinged lid (green) of a Ca_V_1.3 channel. Note the absence of palmitoylation of this β subunit which allows the VDI hinged lid to move freely. (**E**) Exemplar Ba^2+^ current trace through WT (black) and A760G (blue) Ca_V_1.3_short_ channels in the presence of β_1b_. Note the absence of VDI in WT channels despite the lack of β subunit palmitoylation due to the presence of a VDI shield. A760G causes a pronounced re-emergence of VDI under these conditions. (**F**) The increase in VDI is confirmed in population data where Ba^2+^ *r*_300_ values for WT (black) and A760G (blue) are plotted as a function of voltage. At 10 mV, WT: *r*_300_ = 0.98 ± 0.01, n = 3; A760G: *r*_300_ = 0.77 ± 0.05, n = 4. VDI is significantly increased for A760G channels (p < 0.01).

In addition to the critical Ca^2+^-dependent feedback, VDI also plays a major role in controlling Ca^2+^ entry through Ca_V_1 channels. The underlying structural components for VDI have been shown to involve the linker region between domains I and II (I-II linker), which acts as a hinged-lid to close the pore following depolarization^14^,^48^. This process is known to be variably modulated by the binding of different isoforms of channel β subunits to the I-II linker^14^,^26^. For example, when coexpressed with β_2a_, Ca_V_1 channels display little VDI due to restricted movement caused by palmitoylation, and thus membrane anchoring, of the β_2a_ (Fig. 4A, blue)^28^,^51^,^52^. On the other hand, if the channels are coexpressed with the β_1b_ subunit, most Ca_V_1 isoforms will display strong VDI due to the lack of the palmitoylation site^22^,^28^. Ca_V_1.3 channels, however, are unique in that their S6 helices have been shown to act as a shield (Fig. 4A,D, red) to prevent closing of the I-II linker lid, thus, endowing the channels with minimal VDI regardless of β subunit isoform co-expressed.

A close inspection of the Ba^2+^ current through the A760G Ca_V_1.3 channels reveals a slight, but significant re-emergence of VDI despite the presence of the β_2a_ subunit (Fig. 4B,C). Importantly, this mutation-induced VDI can be discerned in either of the two channel variants (Fig. 1G versus B, Fig. 3F versus A). This re-emergence of VDI is accentuated when A760G Ca_V_1.3 is coexpressed with β subunits lacking a palmitoylation site. Figure 4E,F demonstrate a significant increase in VDI in channels harboring the A760G mutation in the presence of the β_1b_ subunit. Note that the WT Ca_V_1.3 channels (black) have minimal VDI even in the presence of the β_1b_ subunit due to the existence of the ‘shield’. Thus the re-emergence of VDI in these channels indicates that the A760G mutation may disrupt the VDI shield (Fig. 4A,D, bottom). This increase in VDI may act in opposition to the loss of CDI within these mutant channels, thus mitigating the detrimental effects of increased Ca^2+^ flux into cells.

### The A760G mutation can cause an increase in intracellular Ca^2+^

We now know that the A760G mutation has a significant effect on CDI, VDI, and deactivation. Given the known link between excess cytosolic Ca^2+^ and severe disease states^39^,^60^,^61^, we wondered if Ca^2+^ overload due to an increased Ca^2+^ influx may play a role in the phenotype of A760G patients. However, the effects of the A760G mutation on Ca^2+^ entry are multi-fold, such that a decrease in CDI and slowing of channel deactivation are opposed by an increase in VDI. We therefore sought to confirm the cumulative effect of the A760G mutation on overall Ca^2+^ entry and intracellular Ca^2+^ concentration ([Ca^2+^]_i_).

**Figure 5:**
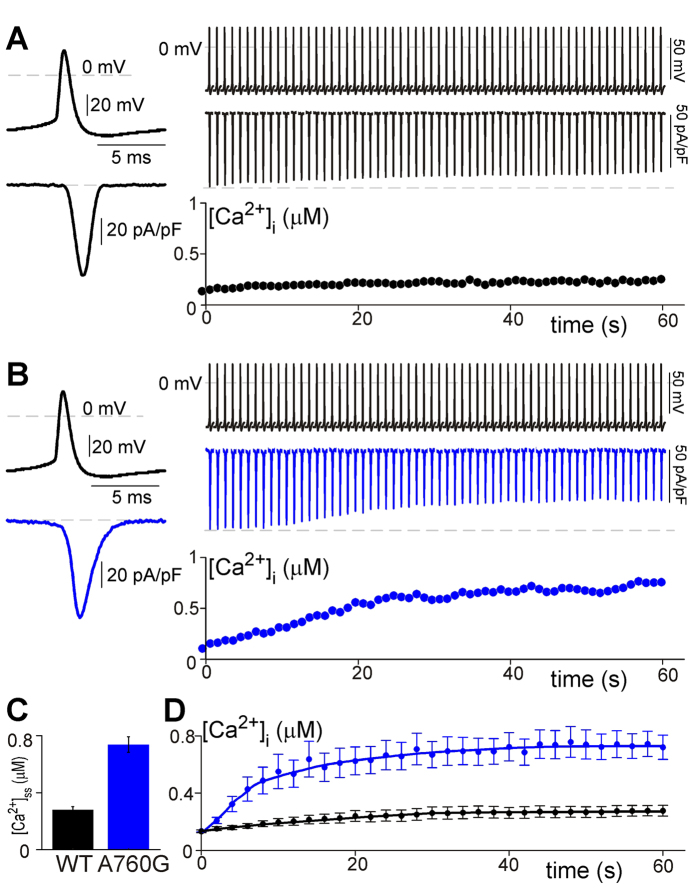
Effects of A760G on cytosolic Ca^2+^. (**A**) Exemplar Ca^2+^ currents in response to a 1-Hz train of neuronal action potentials delivered to HEK293 cells expressing WT Ca_V_1.3_short_ channels. On the left, a single action potential and corresponding Ca^2+^ current is displayed on an expanded time course for resolution. Each action potential (top) and corresponding current response (middle) is magnified for display purposes and represents a 23 ms interval. The peak of each action potential is aligned with the time course displayed on the bottom panel. Note the gradual decrease in peak current as the level of cytosolic Ca^2+^ (bottom right panel) rises. (**B**) Exemplar Ca^2+^ currents in response to a 1-Hz train of neuronal action potentials delivered to HEK293 cells expressing A760G Ca_V_1.3_short_ channels. On the left, a magnified view demonstrates an increased duration of Ca^2+^ entry (blue) during a single action potential as compared to WT (**A**). At a comparable current density, A760G causes considerably more cytosolic Ca^2+^ accumulation (bottom right panel) as compared to WT channels. This increased cytosolic Ca^2+^ persists despite causing an enhancement of CDI (middle right panel). (**C**) Steady state level of intracellular Ca^2+^ in response to a 1-Hz train of action potentials. [Ca^2+^]_ss_ is measured after 60 s of stimulation. A760G Ca_V_1.3 channels display significantly higher levels of [Ca^2+^]_ss_ than WT channels (WT: 0.28 ± 0.05, n = 7; A760G: 0.73 ± 0.13, n = 8; p < 0.01). (**D**) Average [Ca^2+^]_i_ as a function of time as HEK293 cells expressing WT or A760G Ca_V_1.3_short_ channels are stimulated by a train of 1-Hz action potential. Error bars indicate ± SEM, n = 7, 8 for WT and A760G respectively.

To this end, we stimulated HEK293 cells expressing Ca_V_1.3 channels with a 1-Hz train of neuronal action potentials (Fig. 5A, top) and recorded both the Ca^2+^ current passing through the channels as well as the intracellular Ca^2+^ level. This stimulation protocol was chosen as it matches well with the spontaneous neuronal firing frequencies in some populations of hippocampal neurons, thus mimicking a basal activity level^62^,^63^. When this protocol was applied to WT Ca_V_1.3 channels, a small decrease in peak current amplitude was observed over time as CDI accumulated (Fig. 5A, middle panel). This amplitude decay matches well with the relatively small increase in [Ca^2+^]_i,_ (Fig. 5A, bottom panel). When the A760G mutation was introduced into the channel, however, a significant increase in both the rate and extent of intracellular Ca^2+^ accumulation was observed (Fig. 5B–D, blue) due to an increased duration of Ca^2+^ entry during each action potential (Fig. 5B, left). This increased Ca^2+^ entry is likely a net result of decreased channel inactivation and a slowing of channel deactivation. This excess [Ca^2+^]_i_ increased the extent of CDI observed in the current recordings over time, although this decrease in current entry was not sufficient to counteract the Ca^2+^ overload within the cytosol. Overall, the cumulative effect was a steady state Ca^2+^ level approaching 0.8 uM (Fig. 5C), substantially larger than the normal resting [Ca^2+^]_i_ of a neuron^64^. This excess cytosolic Ca^2+^ due to A760G could be a significant contributing factor to the disease pathogenesis.

## Discussion

We have demonstrated a significant effect of the autism-associated mutation A760G on the gating of Ca_V_1.3 such that channel activation is significantly left-shifted, CDI is decreased, and deactivation is slowed, resulting in excess Ca^2+^ entry through channels. However, these effects are mitigated by an increase in VDI. Importantly, the balance between these opposing mechanisms may be dependent on the specific properties of the particular channel complex harboring the mutation. In particular, we have demonstrated that two major Ca_V_1.3 splice variants respond to the introduction of the A760G mutation with distinct levels of CDI disruption (Figs 1 and 3). As each of these splice variants has a significantly different affinity for commonly used L-type channel blockers^59^, such a variation in CDI effects may have important implications on the response of patients to treatment. Moreover, these two splice variants represent only a subset of a panoply of channel isoforms found across different tissues, each with uniquely tuned channel gating and feedback regulation^3^,^11^,^17^,^18^,^19^,^59^. Like the short versus long splice isoforms, it is possible that the A760G mutation may differentially alter the biophysical properties of these variants. Thus the extent of channel alteration may depend on the expression pattern within each specific cell type resulting in variable phenotypes across different tissues.

The original case report for A760G describes a patient exhibiting primarily neurological deficits classified as ASD^30^. Upon further examination of the Simons Simplex Collection database, the classification of ASD in this proband appeared to be of a relatively milder nature on the autism spectrum (pervasive developmental disorder not otherwise specified or PDD NOS) without additional non-neurological symptoms. Such a narrow symptom profile without any cardiac or hearing deficits stands in contrast to the broad tissue distribution of Ca_V_1.3. Moreover, this lack of severe multisystem characteristics is unusual for autism-related Ca^2+^ channelopathies^29^,^39^,^40^,^65^. The multitude of Ca_V_1.3 channel variants across different tissues^17^,^22^,^23^ may account for some of this lack of a multi-system phenotype. The relatively moderate symptoms of this proband may be due, in part, to differential effects of the A760G mutation on specific channel variants expressed in each system. In addition, the VDI effects of the A760G mutation could also contribute to the milder phenotype displayed by the proband. In particular, the increased Ca^2+^ entry due to altered CDI and channel activation/deactivation may be partially offset by increased VDI. As this VDI enhancement is accentuated in the presence of select beta subunits (Fig. 4), expression patterns of different beta subunits^66^ may further increase the variability of A760G effects across systems.

The A760G mutation is capable of substantially raising cytosolic Ca^2+^ concentration when overexpressed in HEK293 cells (Fig. 5). Of note, this effect was achieved even at a relatively slow 1-Hz pacing rate, comparable to the spontaneous firing rate of some hippocampal neurons^62^,^63^. The experimental conditions utilized here were optimized for maximal resolution of the A760G effect. However, under physiological heterozygous expression levels, the effect of the A760G channels will likely be considerably less. Nonetheless, the idea of excessive Ca^2+^ entry underlying ASD is not unprecedented as the ASD phenotype has been linked to Ca^2+^ overload through a myriad of Ca^2+^ handling molecules^67^,^68^,^69^, including multiple voltage-gated Ca^2+^ channels^39^,^40^,^65^,^70^. Overall, it seems plausible that the gating defects of the Ca_V_1.3 channels harboring the A760G mutation may result in excess Ca^2+^ entry, which in turn may be over-activating the downstream Ca^2+^ signaling pathways involved in neural development and plasticity^67^,^68^,^69^,^70^. While the mechanisms underlying ASD remain elusive, the identification of mutations such as A760G, hint at important contributing factors.

## Methods

### Molecular Biology

The point mutation (A760G) was introduced into rat Ca_V_1.3 short and long splice variants (gifts from Dr. Tuck Wah Soong^59^) in the homologous position to that found in humans using QuikChange™ site-directed mutagenesis (Agilent). The equivalent human mutation was found in patients at A769G in chromosome 3, position 53764493^30^ and corresponds to the A749G mutation previously described in an alternate human splice variant^31^.

### Transfection of HEK293 cells

HEK293 cells were cultured on glass coverslips in 10-cm dishes and WT or mutant Ca_V_1.3 channels, along with their auxiliary subunits, were transiently transfected using a standard calcium phosphate method^71^. 8 μg of rat Ca_V_1.3 was co-expressed with 8 μg of rat brain β_2a_ (M80545) or β_1b_ (NM_017346), 8 μg of rat brain α_2_δ (NM012919.2) subunits, and 2 μg of simian virus 40 T antigen cDNA. Expression of all constructs was driven by a cytomegalovirus promoter and β subunits were contained within an EGFP-IRES bicistronic vector to allow visualization of transfected cells.

### Whole Cell Electrophysiology

Whole-cell voltage-clamp recordings of HEK293 cells were done 1–2 days after transfection at room temperature. Recordings were obtained using an Axopatch 200B amplifier (Axon Instruments). Whole-cell voltage-clamp records were low pass filtered at 2 kHz, and then digitally sampled at 10 kHz. P/8 leak subtraction was used, with series resistances of 1–2 MΩ. For voltage-clamp experiments, internal solutions contained (in mM): CsMeSO_3_, 114; CsCl, 5; MgCl_2_, 1; MgATP, 4; HEPES (pH 7.3), 10; and either BAPTA, 10 or EGTA, 0.5; at 295 mOsm adjusted with CsMeSO_3_. External solutions contained (in mM): TEA-MeSO_3_, 140; HEPES (pH 7.4), 10; and CaCl_2_ or BaCl_2_, 40; at 300 mOsm, adjusted with TEA-MeSO_3_.

For simultaneous ratiometric Ca^2+^ measurements and current recordings, a fixed ratio of two Ca^2+^-sensitive dyes (Fluo-2 high affinity, TEFLabs; Fluo-2 low affinity, TEFLabs) and Alexa568 (Invitrogen) were added into a 0.5-mM EGTA internal solution. Two Ca^2+^ indicators at different binding affinity were chosen to accurately measure both baseline and peak Ca^2+^ concentrations. The dye mixture was calibrated to obtain absolute Ca^2+^ concentrations^72^. The external solution (Tyrode’s solution) contained (in mM): NaCl, 135; KCl, 5.4; CaCl_2_, 1.8; MgCl_2_, 0.33; NaH_2_PO_4_, 0.33; HEPES, 5; glucose, 5 (pH 7.4). During current recordings, Ca^2+^ concentration was measured by exciting dyes using a 514-nm Argon laser, via a 545DCLP dichroic mirror and either a 545/40BP (Fluo) or 580LP (Alexa568) filter. Cells were held at −80 mV and a 1-Hz train of neuronal action potential recordings from cortical neurons of E18 mouse embryos were used as voltage stimulus.

### Generation of a Ca_V_1.3 Homology Model

We used MODELLER v9.14^73^ to build a homology model of Ca_V_1.3 pore regions (Fig. 1A) based on the crystal structure of the bacterial Na channel Na_V_Ab (PDB accession code: 4EKW)^74^ as previously described^14^. Briefly, we generated 10 decoy models from which a model with lower objective function was chosen. The four domains of Ca_V_1.3 were constrained to adopt a clockwise orientation when viewed from the extracellular surface by analogy to the orientation of the related voltage-gated sodium channels^75^. The alignment used for the various pore subsegments are as follows:

*S5 Segment*

Na_V_Ab SVAALLTVVFYIAAVMATNLYGATFP

Ca_V_1.3 Domain I HIALLVLFVIIIYAIIGLELFIGKMH

Ca_V_1.3 Domain II SLLLLLFLFIIIFSLLGMQLFGGKFN

Ca_V_1.3 Domain II INIMIVTTLLQFMFACIGVQLFKGKFY

Ca_V_1.3 Domain IV YVALLIAMLFFIYAVIGMQMFGKVAM

*P Loop*

Na_V_Ab EWFGDLSKSLYTLFQVMTLESWSMGIVRPVMNV

Ca_V_1.3 Domain I TNFDNFAFAMLTVFQCITMEGWTDVLYWVNDAI

Ca_V_1.3 Domain II STFDNFPQALLTVFQILTGEDWNAVMYDGIMAY

Ca_V_1.3 Domain III FNFDNVLSAMMVLFTVSTFEGWPALLYKAIDSN

Ca_V1_.3 Domain IV NNFQTFPQAVLLLFRCATGEAWQEIMLACLPGK

*S6 Segment*

Na_V_Ab HPNAWVFFIPFIMLTTFTVLNLFIGII

Ca_V_1.3 Domain I WEWPWVYFVSLIILGSFFVLNLVLGVL

Ca_V_1.3 Domain II GMIVCIYFIILFICGNYILLNVFLAIA

Ca_V_1.3 Domain III RVEISIFFIIYIIIVAFFMMNIFVGFV

Ca_V_1.3 Domain IV SNFAIVYFISFYMLCAFLIINLFVAVI

### Data Analysis and Statistics

The fraction of current remaining after 300 ms of channel activation (*r*_300_) was calculated as:


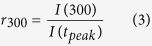


and CDI measurements were corrected for VDI effects by calculating a metric for pure CDI (*f*_300_) as follows:


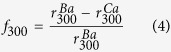


*r*_300_ and *f*_300_ values were reported at 10 mV for the Ca_V_1.3_short_ channel and at 0 mV for Ca_V_1.3_long_. The relative open probability (*P*_O,rel_) was determined by a tail activation protocol^54^ where channels are fully activated at 80 mV prior to stepping to variable test potentials. The ratio of peak and steady state currents then represent the relative *P*_*O,rel*_ of each voltage. The voltage activation curve was fit by the Boltzmann equation:


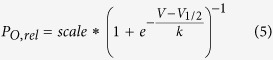


where *V*_1/2_ and *k* represent the half activation voltage and slope factor, respectively.

Time constants for channel deactivation (τ_fast_ and τ_slow_ in Figs 1F,K and 3E,J) were calculated by fitting the deactivating Ba^2+^ tail currents resulting from a transition from 80 mV (channels maximally open based on the activation curve, Figs 1D,I and 3C,H) to multiple voltages near the base of the activation curve (channels closed) with the equation:


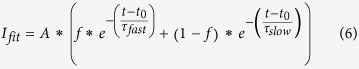


where *f* is the fraction of the faster portion of the current decay, and time constants τ_fast_ and τ_slow_ represent the fast and slow components of the current decay.

All data are presented as mean ± SEM. Statistical significance for variability was determined by a two-tailed student’s t-test.

## Additional Information

**How to cite this article**: Limpitikul, W. B. *et al.* An autism-associated mutation in Ca_V_1.3 channels has opposing effects on voltage- and Ca^2+^-dependent regulation. *Sci. Rep.* **6**, 27235; doi: 10.1038/srep27235 (2016).

## Supplementary Material

Supplementary Information
