# Supplementary material for: An autism-associated mutation in CaV1.3 channels has opposing effects on voltage- and Ca2+-dependent regulation
Source: Sci Rep. 2016 Jun 3;6:27235. doi: 10.1038/srep27235 (PMC4891671; doi:10.1038/srep27235)
Supplement: Supplementary Information [file srep27235-s1.pdf]

Supplementary Information

An autism-associated mutation in $Ca_V1.3$ channels has opposing effects on voltage- and Ca^{2+}- dependent regulation

Worawan B. Limpitikul[1,‡], Ivy E. Dick[1,‡], Manu Ben-Johny[1], David T. Yue[1]

[1]Calcium Signals Laboratory
Departments of Biomedical Engineering and Neuroscience
The Johns Hopkins University School of Medicine
Ross Building, Room 713
720 Rutland Avenue
Baltimore, MD 21205
voice: (410) 955-0079
fax: (410) 955-0549
email: wlimpit1@jhmi.edu; ied@jhu.edu

[‡]To whom correspondence should be addressed

Table of Contents

Supplementary Information 1	Additional Data
Supplementary Information 2	Supplementary References

1. Additional Data

1.1 The residual inactivation of A760G $Ca_V1.3$ remains Ca^{2+}/CaM dependent

CDI of Ca_V channels is known to be orchestrated by the Ca^{2+} sensor molecule calmodulin (CaM)[1-3]. To ensure that the residual inactivation of Ca^{2+} current observed in $Ca_V1.3$ channels harboring the A760G mutation remains entirely dependent on Ca^{2+}/CaM, we co-expressed the mutant channel with a dominant negative mutant CaM (CaM_{1234})[4], incapable of binding Ca^{2+}. The A760G $Ca_V1.3_{short}$ channels displayed no CDI (Supplementary Figure 1), confirming that the A760G mutation did not alter the mechanism underlying CDI of these channels. Gratifyingly, this Ca^{2+}/CaM dependence is identical to that seen in WT $Ca_V1.3$ channels co-expressing with CaM_{1234}[5].

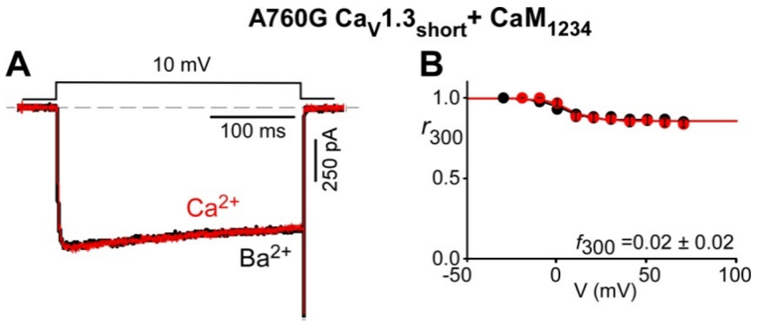

Supplementary Figure 1. Elimination of CDI of A760G $Ca_V1.3$ upon co-expression of CaM_{1234}. (A) Exemplar Ca^{2+} (red) and Ba^{2+} (black) currents from $Ca_V1.3_{short}$ channels harboring the A760G mutation with CaM_{1234} overexpressed. Scale bar corresponds to Ca^{2+} trace. Ba^{2+} trace is normalized to Ca^{2+} for comparison. (B) Population data of Ca^{2+} (red) and Ba^{2+} (black) current remaining after 300 ms (r_{300}). Error bars indicate ± SEM.

1.2 A760G decreases CDI_{max} of $Ca_V1.3$ at multiple voltages

In the main text, we illustrate a reduction in CDI_{max} at a 10-mV test potential (main text Figure 2). To account for the shift in channel activation produced by the A760G mutation, we also quantified CDI_{max} of both WT and A760G $Ca_V1.3_{short}$ at 0-mV and 20-mV test potentials (Supplementary Figure 2). CDI_{max} of the WT channels is significantly higher than that of the A760G channels at each voltage tested, confirming a reduction of CDI_{max} in A760G channels across voltages.

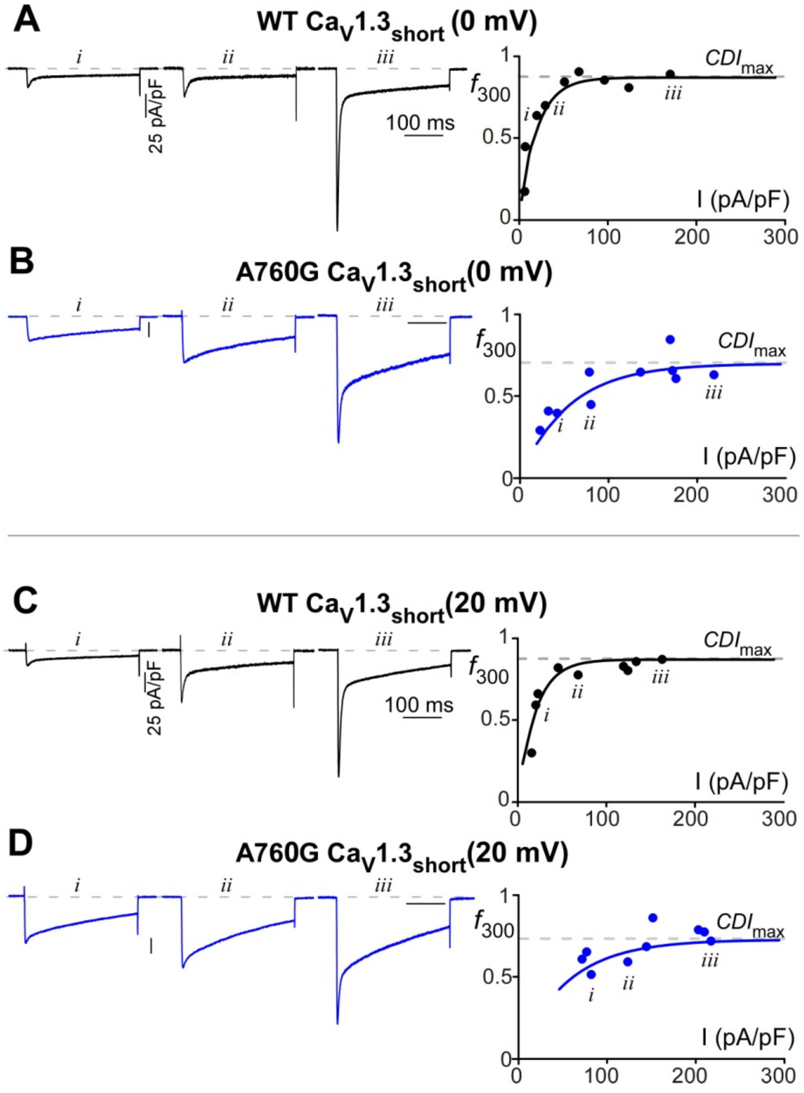

Supplementary Figure 2. Reduction of CDI_{max} at various voltages. (A) Ca^{2+} currents of WT $Ca_V1.3_{short}$ obtained under low Ca^{2+} buffering (0.5 mM EGTA) evoked by a 0-mV test potential. The extent of CDI increases as a function of current density and reaches a maximal value of ~ 0.9. (B) Ca^{2+} traces from A760G $Ca_V1.3_{short}$ obtained in a similar manner as 2A. The extent of CDI increases as the current density increases and reaches a CDI_{max} of ~ 0.7, significantly lower than that of the WT channel. (C-D) Ca^{2+} currents through WT and A760G $Ca_V1.3_{short}$ channels evoked by a 20-mV test potential under 0.5-mM EGTA buffering. At this test potential, CDI_{max} of the WT channels (~ 0.9) remains consistently higher than that of the A760G channels (~ 0.75).

2. Supplementary References

1 Ben-Johny, M. & Yue, D. T. Calmodulin regulation (calmodulation) of voltage-gated calcium channels. *J. Gen. Physiol.* **143**, 679-692, doi:10.1085/jgp.201311153 (2014).

2 Halling, D. B., Aracena-Parks, P. & Hamilton, S. L. Regulation of voltage-gated Ca2+ channels by calmodulin. *Sci STKE* **2006**, er1 (2006).

3 Christel, C. & Lee, A. Ca2+-dependent modulation of voltage-gated Ca2+ channels. *Biochim. Biophys. Acta* **1820**, 1243-1252, doi:10.1016/j.bbagen.2011.12.012 (2012).

4 Xia, X. M. *et al.* Mechanism of calcium gating in small-conductance calcium-activated potassium channels. *Nature* **395**, 503-507, doi:10.1038/26758 (1998).

5 Yang, P. S. *et al.* Switching of Ca2+-dependent inactivation of Ca(v)1.3 channels by calcium binding proteins of auditory hair cells. *J. Neurosci.* **26**, 10677-10689, doi:10.1523/JNEUROSCI.3236-06.2006 (2006).
